# iMapSplice: Alleviating reference bias through personalized RNA-seq alignment

**DOI:** 10.1371/journal.pone.0201554

**Published:** 2018-08-10

**Authors:** Xinan Liu, James N. MacLeod, Jinze Liu

**Affiliations:** 1 Department of Computer Science, University of Kentucky, Lexington, KY, United States of America; 2 Department of Veterinary Science, University of Kentucky, Lexington, KY, United States of America; Boston University, UNITED STATES

## Abstract

Genomic variants in both coding and non-coding sequences can have functionally important and sometimes deleterious effects on exon splicing of gene transcripts. For transcriptome profiling using RNA-seq, the accurate alignment of reads across exon junctions is a critical step. Existing algorithms that utilize a standard reference genome as a template sometimes have difficulty in mapping reads that carry genomic variants. These problems can lead to allelic ratio biases and the failure to detect splice variants created by splice site polymorphisms. To improve RNA-seq read alignment, we have developed a novel approach called iMapSplice that enables personalized mRNA transcriptome profiling. The algorithm makes use of personal genomic information and performs an unbiased alignment towards genome indices carrying both reference and alternative bases. Importantly, this breaks the dependency on reference genome splice site dinucleotide motifs and enables iMapSplice to discover personal splice junctions created through splice site polymorphisms. We report comparative analyses using a number of simulated and real datasets. Besides general improvements in read alignment and splice junction discovery, iMapSplice greatly alleviates allelic ratio biases and unravels many previously uncharacterized splice junctions created by splice site polymorphisms, with minimal overhead in computation time and storage. Software download URL: https://github.com/LiuBioinfo/iMapSplice.

## Introduction

In the past decade, high throughput sequencing (HTS) has been established as one of the major technologies to investigate the genome, epigenome, and transcriptome from tissue samples or even a single cell. Besides its unprecedented resolution due to deep sequencing, one of the great advantages of HTS is that it does not rely on any prior knowledge of sequence in sampling the genomic content from any given subject, which is critical for the discovery of previously uncharacterized genomic features. Recognizing these opportunities, large studies such as The Cancer Genome Atlas (TCGA, (http://cancergenome.nih.gov/) [1,2] and the 1000 genomes project [3] have generated both genomic data in the form of whole genome or whole exome sequencing and transcriptomic data in the form of mRNA sequencing (RNA-seq). Recently, an approach called G&T-seq has been developed and is capable of performing parallel sequencing of both the genome and transcriptome from a single cell [4]. These datasets are often generated to study the relationships between genetic variation and the transcriptome among individuals or even between specific cells. In the meantime, the availability of these multi-omics data presents an opportunity for integrative analysis, where information from different datasets can be borrowed to enhance the performance with the commonly used single-omics analysis.

This paper studies whether and how SNPs called from DNA-seq of a given subject can be used to perform more accurate alignment of RNA-seq reads from that same individual. As we mentioned above, sequencing is a sampling process that is subject-specific and priori knowledge-independent. Unfortunately, a level of bias can be introduced if RNA sequencing data are aligned to a standard reference genome. Although the current state-of-the-art spliced aligners already perform quite well in the discovery of previously unannotated splice junctions and even some structure variations, they almost always utilize the reference genome of a species as the template for read alignment [5–10]. The consequence of the reference-based approach is that the alignment will favor sequences with the highest identities to the reference genome. While this assumption ensures the accurate alignment of a vast majority of the sequencing reads, it can introduce bias against a special category of reads that deviate slightly from the reference but have the potential to be biologically significant in a specific subject. Besides noise and errors, this set of reads is often attributed to single nucleotide polymorphisms (SNPs), small indels, as well as splice variants as a result of splice site polymorphisms. The inability to accurately align this set of reads is often referred to as reference bias [11]. Its effect in generating false positives in genotype calls as well as allele frequency estimations has been noted in several studies involving whole genome and exon sequencing [11,12]. With RNA-seq, this reference bias can lead to a deficiency in characterizing transcripts carrying alternate alleles instead of the reference, compromising the identification of allele-specific transcripts that may be critical to characterizing various biological phenomena such as cis-regulatory variation and nonsense-mediated decay [13].

Additionally, splice site polymorphisms, where non-canonical splice site can be converted into canonical splice sites (containing canonical dinucleotides GT-AG, GC-AG, or AT-AC, see S6 Table for a complete list of canonical/noncanonical splice site dinucleotides), are associated with the expression of alternative transcript variants [14]. Such alterations in the transcriptome have the potential to either cause disease directly or contribute to the susceptibility or severity of disease [15–17]. However, most of the RNA-seq aligners rely on the canonical flanking bases (e.g. GT-AG, GC-AG, and AT-AC) in the reference genome to perform confident spliced alignments. As such, variant transcripts in a specific individual associated with splice site polymorphisms at non-canonical splice sites can be substantially penalized unless this parameter is completely disabled in the mapping software [18].

A straightforward approach to solve bias introduced by the reference genome would be to use personalized genomes, where the specific nucleotide sequence of each individual subject is approximated by substituting SNPs at the corresponding reference coordinates. This strategy was adopted by rPGA [14] where, in addition to the reference genome, the reads are also mapped to the subject's two haplotype genomes. Such a strategy triples the amount of disk space and running time required when using a single genome in both indexing and mapping steps. To perform read alignment to a genome, the first step is to build an index for it, which is always very time-consuming. In addition, indexing files always consume five to tens of gigabytes in storage for each genome. Taking rPGA as an example, although it leverages the super-fast aligner STAR [10] as its backbone, it still takes an average of 4.3 CPU hours to index the three genome versions (one standard reference genome and two haplotype genomes) for each subject. At the same time, storing those indexing files requires about 79 GB disk space. This does not even count the time and space consumptions of extra post processing steps necessary to merge individual haplotype's alignment results into one consensus alignment. Taken together, the computational requirements raised by mapping to subject genomes would greatly limit its efficiency when aligning datasets involving hundreds or even thousands of individuals. Another possible approach would be to use a graph-based index. The graph-based indexing approach is mainly designed to enable read alignments against a large collection of genomes [19,20]. Different from the standard linear-based approach, which is only able to index a single genome, it represents a collection of genomes with a graph. It would be space- and time- efficient to build a single graph-base index for all the individuals’ genomes and use it as the reference template to map all the RNA-seq reads. However, this would introduce another source of error since reads could potentially be aligned to the other individuals’ genomes. Further, if we build the graph-based index for each individual separately, it will suffer the same problem of computational burden as the first strategy (adopted by rPGA).

In this paper, we propose a new approach for individualized RNA-seq alignment, designated iMapSplice. It makes use of personal genomic information and performs an unbiased alignment towards a genome index carrying both the reference and any alternative bases. The approach is light-weight and does not require a whole genome index to be rebuilt for each individual. Importantly, it breaks the computational emphasis or dependency on the position of canonical splice site dinucleotide motifs in the reference genome and enables iMapSplice to discover personal splice junctions created through splice site polymorphisms. We report comparative analyses using a number of simulated and real human datasets. The results demonstrate improvements in general read mapping, accurate alignment yields, and both the sensitivity and accuracy of splice junction discovery. At the same time iMapSplice greatly reduces the biases in reference allelic ratio and discovers many personal splice junctions.

## Methods

iMapSplice extends the MapSplice algorithm [9] and achieves the goal of personalized alignments through the usage of individual genomic information. In brief, MapSplice operates in three steps to align RNA-seq reads onto the reference genome and detect splice junctions. It first searches for exonic mapping of read segments onto the reference genome. Then, the adjacent mapped read segments will be bridged through spliced alignment if they are located separately on the reference genome. In the last step, all of the read segments are assembled to create candidate read alignments and the best one will be reported as the final alignment of reads. In each of these steps, iMapSplice adopts various strategies to utilize data provided from genomic DNA single nucleotide variants. It is able to recover read alignments that either harbor SNPs or contain spliced alignments flanked by splice sites with polymorphisms. This section provides an overview of how the method works to address the challenges faced by mapping reads only to a reference genome.

**Fig 1:**
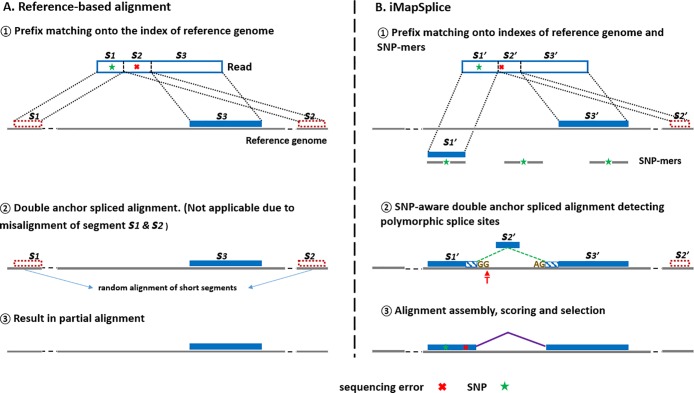
An overview of iMapSplice algorithm. (A) An example illustrating the challenge when mapping a RNA-seq read to the reference genome in the presence of SNPs. (B) An example illustrating how iMapSplice algorithm may resolve spliced alignment with SNPs as well as the basic steps of the alignment.

The left panel in Fig 1 illustrates an example of how an RNA-seq read carrying a SNP may fail to align correctly to a reference genome. The RNA-seq read in this example carries a SNP as well as a sequencing error. One of the general strategies used in the current fast aligners is iterative maximal prefix match [8,10]. When searching for a prefix carrying the SNP against the reference, the correct mapping location will be missed because no error is tolerated in this step. More often than not, short prefix alignments (<18bp) to random places (such as *S*_1_ and *S*_2_) are likely to be returned and have to be filtered out. Eventually, this may result in a partial alignment (*S*_3_).

iMapSplice resolves this issue by including knowledge of the SNPs in each alignment step, as shown in the right panel of Fig 1. The first step of iMapSplice searches for the exonic mapping of read segments through an approach called semi-maximal prefix match. Different from a maximal prefix search, in which only the mapping location of the longest match is returned, a semi-maximal prefix search returns all mapping positions with a match longer than a certain threshold (set as 30bp by default). In this step, it simultaneously maps reads to both reference genome as well as the exonic regions harboring by SNPs, namely SNP-mers. A SNP-mer corresponds to a segment of genomic sequence carrying the variant nucleotide, localizing it to the middle of the sequence.

### SNP-mer generation

SNP-mers can be derived in two ways corresponding to two variants of iMapSplice: iMapSplice-phased and iMapSplice-unphased. iMapSplice-phased applies when the phased genotype data are available (such as in the 1000 genomes project). In this case, SNP-mers of a fixed length *k*_phased_ (201bp by default) from the two genomic haplotypes are extracted. The reference nucleotides are then replaced with alternate nucleotides at all of the SNP positions. However, iMapSplice-phased cannot be used when the phase information is not available, in which cases iMapSplice-unphased will be applied.

In iMapSplice-unphased, a combination of variable length SNP-mers are used. Let *k*_max_ (201bp by default) and *k*_min_ (31bp by default) denote the maximum and the minimal length of a SNP-mer, respectively. In extracting the SNP-mer for each SNP, there are three scenarios according to the distance *d* between each SNP and its nearest neighbor SNP. When *d* > *k*_max_, a SNP-mer of length *k*_max_ is extracted; when *k*_max_ ≥ *d* ≥ *k*_min_, a SNP-mer of length *d* is extracted. In the two cases above, except for the specific SNP, all of the other bases are exactly the same as in the reference genome since there are no other SNPs within the genomic window where the corresponding SNP-mer covers. However, when *d* < *k*_min_, there is more than one SNPs within the *k*_min_ window size. This indicates a SNP-rich region. In such cases, if there are *n* SNPs within this window and one of them is the SNP that all SNP-mers are centered upon, the rest are the *n-*1 SNPs within the SNP-mer region. These additional SNPs increase the variety of the SNP-mers. Since the variant in the middle is set, there would be 2^n-1^ possible nucleotide base combinations for the other n-1 SNPs, all these are different from reference. iMapSplice-unphased will randomly select *m* of them. The selection of *m* value affects the performance of iMapSplice-unphased on mapping reads covering this SNP. A small *m* may miss the perfect SNP-mer which matches read sequences without any error. However, a large *m* could confuse the aligner as too many possibilities are provided and also increase the running time. However, such SNP-rich regions are rare, and thus have little impact on the overall performance. S1 Table lists the distributions of SNPs covered by each SNP-mer of different lengths (from 21bp to 101bp) for a randomly selected individual NA12749. For all the SNP-mers, excluding the one introducing it, over 90% of them contain no or only one other SNPs and less than 1% contain five or more.

Furthermore, the selection of SNP-mer length related parameters (*k*_phased_ in iMapSplice-phased, *k*_max_ and *k*_min_ in iMapSplice-unphased) also affects how well iMapSplice works. A SNP-mer has to be long enough to allow a partial read segment to be confidently mapped. However, a SNP-mer too long is not necessary, as they will repeat the exact sequence from the reference genome. To investigate this relationship, we conducted an experiment to study the impact of SNP-mer length on iMapSplice-phased performance. The results and a more extensive discussion of SNP-mer length selection strategy can be found in S1 File, section 2 and S2 Table.

Enhanced suffix array [21] based indices of SNP-mers are built to facilitate the exonic alignment step (prefix match). Enhanced suffix array is a specific implementation of suffix array with additional tables. While many suffix array implementations only have one or some of the functions of suffix tree, enhanced suffix array can fully replace suffix tree in a space-efficient manner. Approximately 120,000 exonic SNPs were called for each individual human genome according to the 1000 genomes project study (see S3 Table). Sequence extraction and index building can be completed in less than one minute, resulting in minimum overhead relative to alignment. iMapSplice performs an iterative semi-maximal prefix match of read sequences against both the reference genome and SNP-mers. Segments mapped to SNP-mers will be converted to the reference genome coordinates and will be combined with other segments mapped to the reference genome.

Next, read mapping segments that are adjacent to each other on the genome will be merged. For two or more segments that are next to each other in the reads, but are separated on the reference genome, a spliced alignment is performed to bridge the two segments. We call this a double anchor spliced alignment. In this step, many spliced aligners rely on the presence of canonical splice site dinucleotides (e.g. GT-AG, GC-AG, and AT-AC) in the reference genome to detect splice junctions. However, a canonical splice site with subject-specific SNPs will go undetected as its reference counter-part is non-canonical and cannot be recognized by reference-based aligners. Even though some aligners are capable of reporting non-canonical splice junctions, high penalties are given to their alignments to avoid false positives [18]. To solve this problem, iMapSplice utilizes the information provided by nucleotide variants in the target region (from a hash table of SNPs) to create a list of candidate canonical splice sites to help in the determination of correct splice site pairings and improve read mapping accuracy. For example, in the second step shown in Fig 1B, with the known SNP (G > T at the donor site), iMapSplice identifies the novel canonical donor splice site (GG > GT) and completes the spliced alignment of segment *S*_2’_. In conclusion, the SNP-aware double anchor alignment will utilize the SNP information to identify personal spliced alignments that otherwise would be either missed or identified as non-canonical splice junctions.

The last step of iMapSplice completes segment assembly, candidate alignment scoring, and selection. In this step, aligned segments are assembled and different candidate alignments are generated with different potential combinations of segments. Candidate alignments are then scored based on the total number of mismatches, spliced alignment, and mapped length. iMapSplice will only report the alignment with the highest score. If multiple alignments have the same highest score, all of them will be reported. One of the most important metrics when scoring is the number of mismatches for each alignment. iMapSplice removes the mismatches that can be attributed to SNPs.

## Results

In this section, we report the performance of iMapSplice with regard to its capability for unbiased alignment of reads harboring SNPs and for the discovery of splice junctions with polymorphic splice sites.

### Datasets and setup for the experiments

Performance was assessed using two types of data: one based on simulated RNA-seq reads and the other using real human datasets.

Simulated datasets: Simulated RNA-seq reads were generated by BEERS [22] with two different variant and error profiles. The low error reads were generated assuming a substitution frequency of 0.001, indel frequency of 0.0005, and base error frequency of 0.005. Corresponding rates in the high error reads were increased fivefold, 0.005, 0.0025, and 0.025 respectively. For both error categories, we generated two RNA-seq datasets with different read lengths, 50bp and 100bp. Each dataset contained 20 million paired-end reads with the same insert length of 200 bp. Note that the simulated data did not contain polymorphic splice sites and thus it cannot be used to assess the discovery of personal splice junctions as a result of splice site polymorphisms. Details of the simulated data can be found in S1 File, section 3.

Real datasets: 74 randomly selected RNA-seq datasets and their corresponding genotypes were downloaded from the Geuvadis RNA sequencing project [23] and the 1000 Genomes Browser [3] (See S3 Table for detailed information). Numbers of reads in the RNA-seq datasets ranged from 44.6 million to 75.8 million. Approximately 3.6 million SNPs were detected for each individual, with roughly 120,000 of them localized to the exonic regions according to human Gencode annotation (release 19) [24]. All of the RNA-seq reads in the real datasets are paired-end reads and 75 base pairs in length.

To assess the performance of iMapSplice, we compared it to a number of publicly available RNA-seq aligners. All of the methods, version information, and the indices used are listed in Table 1. Default settings were used for all parameters.

**Table 1:** The set of tools and their index configuration used in performance comparison.

Category	Method	Version	Index
iMapSplice	iMapSplice-phased	1.0 Beta	reference genome (hg19) + individual phased SNPs
	iMapSplice-unphased	1.0 Beta	reference genome (hg19) + individual unphased SNPs
Ref-based	MapSplice[9]	3.0 Beta	reference genome (hg19)
	HISAT2[18]	2.0.5	reference genome (hg19)
	STAR (2-pass)[10]	2.5.1	reference genome (hg19)
Mask-based	MapSplice MASK	3.0 Beta	reference genome (hg19) + SNP loci masked with Ns
	HISAT2 MASK	2.0.5	reference genome (hg19) + SNP loci masked with Ns
	STAR MASK (2-pass)	2.5.1	reference genome (hg19) + SNP loci masked with Ns
SNP-aware	HISAT2 SNP	2.0.5	reference genome (hg19) + individual unphased SNPs
	HISAT2 POP	2.0.5	reference genome (hg19) + population SNPs
	rPGA[14]	2.0.0	reference genome (hg19) + two haplotype personal genomes

### Improvement in general read mapping

We first assessed improvements of read alignment performance that resulted from incorporating the knowledge of SNPs using the four simulated datasets. In this experiment, we compared iMapSplice-unphased against all the “Ref-based” tools and HISAT2 SNP. iMapSplice-phased and HISAT2 POP are not applicable since the SNP data were not phased and not consistent with the population profile. The “Mask-based” tools and rPGA are not proposed for general read mapping. We collected the numbers of accurate unique alignments reported by each method. As shown in Table 2, iMapSplice-unphased achieved the highest number of accurate unique alignments in each dataset.

**Table 2:** The comparison of five baseline tools in terms of the number of accurate unique alignments out of 20 million synthetic reads in each of the four datasets with different read lengths and error profiles.

Method	Low error 50bp	Low error 100bp	High error 50bp	High error 100bp
iMapSplice-unphased	18,931,227	19,168,761	14,934,484	16,918,697
MapSplice	18,839,058	19,121,804	13,485,776	16,378,977
STAR	18,316,354	18,446,469	13,053,934	14,832,945
HISAT2	17,771,947	18,813,343	9,032,193	11,167,588
HISAT2 SNP	17,965,316	18,810,270	10,190,994	12,501,692

Incorporating SNPs into the index also improved the alignment performance for HISAT2 in three out of the four datasets. Note that the improved alignment percentage varied since it is a function of the percent of SNP affected reads, which is much less in low error data than in high error data. For the subset of simulated reads that achieved an accurate unique alignment with iMapSplice-unphased but failed by MapSplice, we analyzed their alignment status with MapSplice that utilizes the typical method of mapping to reference genome (S4 Table). In general, for short reads in both the low and high error rate categories, the majority of the reads with reads with improved alignment were not mapped at all using the reference genome. Mapping longer reads significantly improved the alignment rate, which is reasonable since the short reads would have been more vulnerable to SNPs especially when partial alignments are allowed. For the longer reads, iMapSplice-unphased improved the accuracy by being better able to get correct unique alignment for many MapSplice reported multi-map reads, as well as completing partial alignments. At the same time, iMapSplice-unphased achieves better performance in splice junction discovery (see S1 File, section 4 for the detailed comparison results). Additionally, to further investigate how the aligners’ performance is impacted by genomic variants and sequencing error frequencies, we applied the aligners to another simulated dataset of 50bp reads with low variant frequency and high sequencing error frequency. The data and results are discussed in S1 File, section 5.

### Reduction of biases in allelic ratio

It is known that mapping reads to a reference genome introduces an overestimation of reference allele frequency and underestimation of non-reference allele frequency [11]. Reads carrying alternative bases at SNP coordinates have an extra mismatch compared to those with the reference nucleotide. In some cases, this extra mismatch would prohibit the aligner from mapping the read correctly, sometimes leading to the whole read being completely unmapped or the end of the read being softclipped because it contains more mismatches than a threshold. If the read is sequenced from repetitive regions in genome, it could also be incorrectly aligned in situations where the sequence in the incorrect mapping position is identical to the read sequence while the correct mapping position has one base difference at the SNP position. One typical approach to reduce the bias is to mask all the SNP positions with “N”s before mapping the reads. Though allelic bias can be eliminated in this way, it also impairs the aligners’ mapping ability as the reads are one more mismatch away from the reference genome. HISAT2 [8] provides another strategy through incorporating both variants and the reference genome into one graph and aligning read sequences to the graph paths. The strategy utilized by iMapSplice to alleviate the reference allelic ratio bias is to introduce the possibility of read mapping onto SNP-mers where alternative alleles are present.

**Fig 2:**
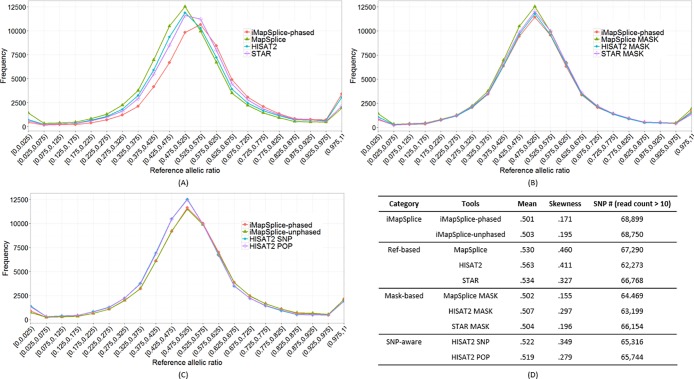
Aggregated reference allelic ratio distribution of different methods on RNA-seq datasets from individuals NA12812, NA12749, NA07056, NA06994, and NA12275. (A) Comparison between iMapSplice-phased and “Ref-based” methods (MapSplice, HISAT2, and STAR); (B) Comparison between iMapSplice-phased and “Mask-based” methods (MapSplice MASK, HISAT2 MASK, and STAR MASK); (C) Comparison between two variants of iMapSplice and “SNP-aware” methods (HISAT2 SNP and HISAT2 POP); (D) Summarized comparison in terms of mean and skewness for reference allelic ratio distributions, and number of SNPs covered by at least ten reads.

Fig 2 shows the aggregated distribution of reference allelic ratio at all SNP positions in randomly selected human RNA-seq datasets from five individuals: NA12812, NA12749, NA07056, NA06994, and NA12275. Compared to the other two category tools, the “Ref-based” programs (in Fig 2A) show significant bias towards the reference allele (reference allelic ratio > 0.5). Obviously, the choice of mapping strategy greatly affects the allelic bias independent of the aligner algorithms themselves. In Fig 2B, both iMapSplice-phased and “Mask-based” methods exhibit symmetric distributions with respect to the reference allelic ratio of 0.5. At the same time, however, iMapSplice-phased delivers the largest number of SNP positions with at least ten supporting reads (see the table in Fig 2D). This is very likely due to the extra mismatches introduced by masking the SNP positions. As reported in Fig 2C, the two variants of iMapSplice perform similarly, and show clear advantages over the two “SNP-aware” variants of HISAT2. These relationships are confirmed in the detailed numerical statistics reported in Fig 2D. Mean and skewness (a measure of the asymmetry of a distribution) [25] are used to characterize the bias for each distribution. iMapSplice-phased and MapSplice Mask achieve the best performances in terms of mean and skewness respectively.

### Discovery of personal splice junctions

We applied iMapSplice-phased to datasets from 74 individuals (See S3 Table for additional information). Detailed personal splice junction detection results are included in S5 Table. The numbers of detected novel canonical splice junctions created by splice site polymorphisms are listed in Table 3. As shown, in total, iMapSplice-phased reported 1,847 personal splice junctions with at least two supporting reads associated with nucleotide changes (polymorphisms) that created a new canonical splice donor and acceptor pair. Among them, 896 and 533 personal splice junctions have at least 5 and 10 supporting reads respectively. Compared with the results reported in another study [14], iMapSplice detected many more polymorphic splice junctions changing from noncanonical to canonical.

**Table 3:** Detected personal splice junctions changing from noncanonical to canonical with increasing numbers of supporting individuals and reads.

Read coverage threshold		Reported by iMapSplice-phased			Previously reported by rPGA[14]		
		≥ 2	≥ 5	≥ 10	≥ 2	≥ 5	≥ 10
**Number of individuals with polymorphic splice sites**	≥ 1	1,847	896	533	506	130	42
	≥ 2	1,698	820	494	229	72	21
	≥ 5	1,615	779	473	119	29	11
	≥ 10	1,499	717	430	63	15	5

We next investigated the effects of splice site polymorphisms on splice junction expression. iMapSplice enabled the discovery of two general types of splice site polymorphisms: (i) the gain of a canonical splice site (e.g. GT-AG, GC-AG, or AT-AC) and (ii) the loss of a canonical splice site. We examined how these polymorphisms affected steady state expression (read counts) at the corresponding splice junctions (Table 4 and Table 5). In this experiment, we categorized all the splice junctions according to changes in average coverage between the individuals with and without splice site polymorphisms.

**Table 4:** Number of splice junctions falling into each category of average coverage changes between the individuals of with and without polymorphisms in splice sites. The polymorphisms changed the splice sites from noncanonical to canonical.

		Average on polymorphic junctions (canonical)			
		0	(0, 2]	(2, 10]	(10, +∞)
**Average coverage on reference junctions (noncanonical)**	0	0	4,482	**210**	**83**
	(0, 2]	30	8	2	**2**
	(2, 10]	1	0	0	0
	(10, +∞)	0	0	0	0

**Table 5:** Number of splice junctions falling into each category of average coverage changes between the individuals with and without polymorphisms in splice sites. The polymorphisms changed the splice sites from canonical to noncanonical.

		Average on polymorphic junctions (noncanonical)			
		0	(0, 2]	(2, 10]	(10, +∞)
**Average coverage on reference junctions (canonical)**	0	0	180	0	0
	(0, 2]	872	37	2	1
	(2, 10]	**10**	4	0	1
	(10, +∞)	**7**	**3**	0	1

Among both types of polymorphic splice junctions, although many had low coverage, iMapSplice-phased detected hundreds of personal splice junctions that exhibited significant expression changes in association with the gain and/or loss of canonical splice site motifs.

For the type (i) polymorphic splice junctions (Table 4), previously non-functional splice sites increased to 3–10 reads at 210 sites and more than 10 reads at 83 sites. In contrast, loss of a canonical splice donor/acceptor pair (ii) significantly inhibited splicing expression (Table 5).

**Fig 3:**
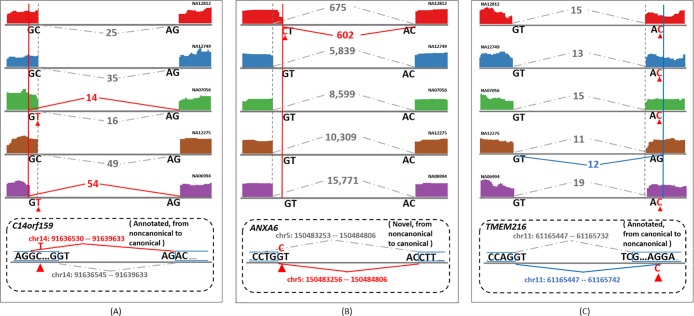
Examples of splice site polymorphisms. The solid lines with numbers indicate splice junctions with a polymorphism in the donor or acceptor site, while the dashed lines with numbers indicate other “normal” splice junctions that share either the donor or acceptor sites with the polymorphic splice junctions, but have no polymorphisms. The vertical solid red lines indicate the splice sites where the polymorphism converts a noncanonical splice site to a canonical site, while the vertical solid blue lines indicate the splice sites where the polymorphism converts a canonical splice site to a noncanonical splice site. The vertical dashed gray lines indicate the unshared splice sites of “normal” splice junctions. Bases in black are the reference nucleotides, while those in red are alternate bases at SNP positions. The numbers along the splice junctions are the supporting read counts in the specific RNA-seq sample. The five RNA-seq samples used to demonstrate the examples are from individuals NA12812, NA12749, NA07056, NA12275, and NA06994. (A) and (B) are examples of splice site polymorphisms that enhance splicing through the creation of canonical splice site dinucleotide; (C) is the example of splice site polymorphism that disables splicing as a result of the loss of canonical splice site dinucleotide.

Polymorphisms in specific splice sites from genes *C14orf159*, *ANXA6*, and *TMEM216* (Fig 3) are examples of the two types of polymorphisms. The polymorphism (C > T) in *C14orf159* (Fig 3A) created a canonical donor site (GC > GT) and led to a novel canonical splice junction in two individuals NA07056 and NA06994. The same splice junction did not show up in RNA-seq data from the other three individuals without this polymorphism. The polymorphism (G > C) in *ANXA6* (Fig 3B) also created a canonical splice site (acceptor site, GT > CT, reverse strand). However, it is different from the one in *C14orf159* that converted an annotated noncanonical splice site to a canonical splice site, it created a completely novel splice junction (previously unannotated). The polymorphism (G > C) in *TMEM216* (Fig 3C) affected expression at the splice junction in the opposite way. This polymorphism corrupted the canonical acceptor site (AG > AC). Four individuals (NA12812, NA12749, NA07056, and NA06994) carrying this nucleotide variant lost the canonical splice junction that appeared in the nonpolymorphic individual (NA12275) with the reference allele.

### Super-fast and space-saving indexing strategy

As mentioned in the Methods section, iMapSplice adopts a very efficient indexing strategy. Compared to a direct approach of rebuilding the index for each personalized genome, this strategy saves a great amount of time and space thereby lowering the risk of computational bottlenecks with large scale applications. iMapSplice takes less than 1 minute to build an index and the indexing files require only around 0.32 Gigabytes in storage for each individual. In contrast, to rebuild the index of an entire personalized genome for each haplotype, rPGA takes approximately 4.3 hours of CPU time creating indexing files that are as large as 79 Gigabytes. Table 6 lists the indexing file storage usage and the runtime of indexing and mapping for iMapSplice on the 74 RNA-seq datasets as well as the estimated results for the other two individual SNP incorporated methods HISAT2 SNP and rPGA. iMapSplice achieved significant advantages in terms of both storage usage and runtime.

**Table 6:** Indexing file size and running time to align the 74 RNA-seq dataset reads. Note: indexing file storage usage and run time for HISAT2 SNP and rPGA are estimated according to their performance on five randomly selected datasets.

Tools	Indexing file storage usage (GB)	CPU Time (hour)		
		indexing	mapping	Total
iMapSplice	90.2	0.6	38.6	39.2
HISAT2 SNP	408	142	14	156
rPGA	5,845	319	44	363

Experiments were run on clusters with nodes equipped with Dual Intel Xeon CPUs E5-26708@2.60GHz and 64GB of 1600MHz RAM.

## Discussion

RNA-seq is a widely adopted technique used in transcriptome profiling for a wide range of applications including differential expression analyses, novel isoform prediction, genomic variants calling, RNA editing, and so on. In most of these applications, especially those that rely on a reference sequence, a critical step is to correctly map each individual RNA-seq read onto the corresponding specific nucleotide coordinates in the reference genome. However, there exists a gap between our current processing methods for RNA-seq data and a fully personalized characterization of an individual’s transcriptome. Genomic DNA sequence differences between individuals are not currently considered in the routine mapping of RNA-seq reads or data analyses. Polymorphic variants such as SNPs may potentially cause the incorrect or incomplete alignment of reads, prohibit the discovery of personal splice junction, and skew expression coverage in the affected regions. As a result, downstream analyses including transcript reconstruction, alternative splicing analysis, and quantitative measurements of transcript expression are compromised. Although statistically they may only affect a small proportion in each category on the whole genome, their functional importance cannot be overlooked as evidenced by existing research [26].

Our evaluation demonstrates that iMapSplice significantly improves the accuracy of RNA-seq read alignment by taking into account both the reference genomic sequence and personal SNP variants. The software performs an unbiased mapping of reads carrying either the reference or alternative base sequence. Comparative results show that the reference allelic ratio distribution derived from the alignment results using iMapSplice exhibits the closest mean relative to 0.5 and the skewness value is also among the smallest ones. Those observations demonstrate that iMapSplice significantly alleviates the reference allelic ratio bias which is a common deficiency for sequencing read aligners. Additionally, SNP variants in an individual can generate novel canonical splice sites dinucleotides or alternatively introduce a base change that alters a canonical splice donor to splice acceptor pairing (GT-AG, GC-AG, or AT-AC). Resulting changes in splice site utilization can be functionally significant and are important to detect. iMapSplice enhances the detection of personal splice sites by considering both reference and individual alternate alleles in determining the optimal alignment for each RNA-seq read.

Performance-wise, iMapSplice is a lightweight approach with minimum overhead in both storage and running time compared to other alignment methods that are also capable of considering individuals SNPs during the mapping process. iMapSplice can be readily applied to the datasets collected in large consortium, such as TCGA, ICGC, as well as the 1000 genomes project by taking either the original reads or the alignment file as input, making it possible to alleviate reference bias and uncover functionally important personalized transcript variants as a result of either polymorphic splice site or allele specific transcript. We expect to continue to improve iMapSplice in the near future to incorporate other structure variations such as small indels. The alignment strategy will be fairly similar and the extension should be straightforward. Also, besides human data, iMapSplice could also be applied to other model species, such as *Arabidopsis thaliana*, *Drosophila melanogaster*, and *Mus musculus*, in the same manner.

Some approaches [8,19] have been proposed to incorporate known common variants (such as those in alternative contigs and dbSNP [27]) into current pipelines in order to improve the alignment performance. Should individual variants not exist, those sub-optimal variants may help when they are consistent with the individual variants. But the most accurate variants for an individual will be those called from the subject’s own genomic data. As sequencing technologies continue to advance, it has become more common to obtain genome sequencing (or SNPs) and RNA-seq data in parallel. Thus we think iMapSplice has the potential to be a widely used computational tool not only for obtaining more reliable read alignments, but also to connect genomic polymorphisms with functionally important variation in splice site utilization. Both of them are indispensable for the characterization of personalized transcriptome and the realization of precision medicine.

## Supporting information

S1 File Supplementary file, including iMapSplice algorithm and usage details, SNP-mer selection and its impact on iMapSplice performance, simulated data information, general splice junction discovery sensitivity and specificity on simulated data, and impact of genomic variant frequency and sequencing error frequency on aligner performance. (DOCX)

S1 Table Distributions of SNPs covered by each SNP-mer of different lengths. (XLSX)

S2 Table The impact of SNP-mer length on iMapSplice-phased in terms of reference allelic ratio distribution. (XLSX)

S3 Table Coriell ids, Geuvadis ids, and SNP numbers of the 74 real human datasets. (XLSX)

S4 Table Read categories correctly aligned by iMapSplice-unphased but failed by MapSplice. (XLSX)

S5 Table Detected splice junctions with polymorphisms at splice sites. (XLSX)

S6 Table Splice site dinucleotide list of canonical/noncanonical splice junctions. (XLSX)

## References

[1] Cancer Genome Atlas Research Network. Comprehensive molecular characterization of clear cell renal cell carcinoma. Nature. 2013;499: 43–9. 10.1038/nature12222 23792563 PMC3771322

[2] Cancer Genome Atlas Research Network. Comprehensive genomic characterization of squamous cell lung cancers. Nature. 2012;489: 519–25. 10.1038/nature11404 22960745 PMC3466113

[3] The 1000 Genomes Project Consortium. A global reference for human genetic variation [Internet]. Nature. 2015 pp. 68–74. 10.1038/nature15393 26432245 PMC4750478

[4] Macaulay IC, Haerty W, Kumar P, Li YI, Hu TX, Teng MJ, et al G&T-seq: parallel sequencing of single-cell genomes and transcriptomes. Nat Methods. 2015;12: 519–522. 10.1038/nmeth.3370 25915121

[5] Trapnell C, Pachter L, Salzberg SL. TopHat: Discovering splice junctions with RNA-Seq. Bioinformatics. 2009;25: 1105–1111. 10.1093/bioinformatics/btp120 19289445 PMC2672628

[6] Wu TD, Nacu S. Fast and SNP-tolerant detection of complex variants and splicing in short reads. Bioinformatics. 2010;26: 873–881. 10.1093/bioinformatics/btq057 20147302 PMC2844994

[7] Kim D, Pertea G, Trapnell C, Pimentel H, Kelley R, Salzberg SL. TopHat2: accurate alignment of transcriptomes in the presence of insertions, deletions and gene fusions. Genome Biol. 2013;14: R36 10.1186/gb-2013-14-4-r36 23618408 PMC4053844

[8] Kim D, Langmead B, Salzberg SL. HISAT: a fast spliced aligner with low memory requirements. Nat Methods. 2015;12: 357–60. 10.1038/nmeth.3317 25751142 PMC4655817

[9] Wang K, Singh D, Zeng Z, Coleman SJ, Huang Y, Savich GL, et al MapSplice: Accurate mapping of RNA-seq reads for splice junction discovery. Nucleic Acids Res. 2010;38 10.1093/nar/gkq622 20802226 PMC2952873

[10] Dobin A, Davis CA, Schlesinger F, Drenkow J, Zaleski C, Jha S, et al STAR: Ultrafast universal RNA-seq aligner. Bioinformatics. 2013;29: 15–21. 10.1093/bioinformatics/bts635 23104886 PMC3530905

[11] Brandt DYC, Aguiar VRC, Bitarello BD, Nunes K, Goudet J, Meyer D. Mapping Bias Overestimates Reference Allele Frequencies at the HLA Genes in the 1000 Genomes Project Phase I Data. G3 (Bethesda). 2015;5: 931–41. 10.1534/g3.114.015784 25787242 PMC4426377

[12] Meynert AM, Ansari M, FitzPatrick DR, Taylor MS. Variant detection sensitivity and biases in whole genome and exome sequencing. BMC Bioinformatics. 2014;15: 247 10.1186/1471-2105-15-247 25038816 PMC4122774

[13] Castel SE, Levy-Moonshine A, Mohammadi P, Banks E, Lappalainen T. Tools and best practices for data processing in allelic expression analysis. Genome Biol. 2015;16: 195 10.1186/s13059-015-0762-6 26381377 PMC4574606

[14] Stein S, Lu ZX, Bahrami-Samani E, Park JW, Xing Y. Discover hidden splicing variations by mapping personal transcriptomes to personal genomes. Nucleic Acids Res. 2015;43: 10612–10622. 10.1093/nar/gkv1099 26578562 PMC4678817

[15] Tazi J, Bakkour N, Stamm S. Alternative splicing and disease. Biochimica et Biophysica Acta—Molecular Basis of Disease. 2009 pp. 14–26. 10.1016/j.bbadis.2008.09.017 18992329 PMC5632948

[16] Zhang F, Wang M, Michael T, Drabier R. Novel alternative splicing isoform biomarkers identification from high-throughput plasma proteomics profiling of breast cancer. BMC Syst Biol. 2013;7 Suppl 5: S8 10.1186/1752-0509-7-S5-S8 24565027 PMC4028860

[17] Ward AJ, Cooper TA. The pathobiology of splicing. Journal of Pathology. 2010 pp. 152–163. 10.1002/path.2649 19918805 PMC2855871

[18] Kim D, Langmead B, Salzberg SL. HISAT: A fast spliced aligner with low memory requirements. Nat Methods. 2015;12: 357–360. 10.1038/nmeth.3317 25751142 PMC4655817

[19] Paten B, Novak AM, Eizenga JM, Garrison E. Genome graphs and the evolution of genome inference. Genome Research. 2017 pp. 665–676. 10.1101/gr.214155.116 28360232 PMC5411762

[20] Kim D, Paggi JM, Salzberg S. HISAT-genotype: Next Generation Genomic Analysis Platform on a Personal Computer. bioRxiv. 2018; 10.1101/266197

[21] Abouelhoda MI, Kurtz S, Ohlebusch E. Replacing suffix trees with enhanced suffix arrays. J Discret Algorithms. 2004;2: 53–86. 10.1016/S1570-8667(03)00065-0

[22] Grant GR, Farkas MH, Pizarro AD, Lahens NF, Schug J, Brunk BP, et al Comparative analysis of RNA-Seq alignment algorithms and the RNA-Seq unified mapper (RUM). Bioinformatics. 2011;27: 2518–2528. 10.1093/bioinformatics/btr427 21775302 PMC3167048

[23] Lappalainen T, Sammeth M, Friedländer MR, ‘t Hoen P a C, Monlong J, Rivas M a, et al Transcriptome and genome sequencing uncovers functional variation in humans. Nature. 2013;501: 506–11. 10.1038/nature12531 24037378 PMC3918453

[24] Harrow J, Frankish A, Gonzalez JM, Tapanari E, Diekhans M, Kokocinski F, et al GENCODE: The reference human genome annotation for the ENCODE project. Genome Res. 2012;22: 1760–1774. 10.1101/gr.135350.111 22955987 PMC3431492

[25] Doane DP, Seward LE. Measuring Skewness: A Forgotten Statistic? J Stat Educ. 2011;19: 1–18. doi: 10.1.1.362.5312

[26] Munger SC, Raghupathy N, Choi K, Simons AK, Gatti DM, Hinerfeld DA, et al RNA-Seq alignment to individualized genomes improves transcript abundance estimates in multiparent populations. Genetics. 2014;198: 59–73. 10.1534/genetics.114.165886 25236449 PMC4174954

[27] Sherry ST. dbSNP: the NCBI database of genetic variation. Nucleic Acids Res. 2001;29: 308–311. 10.1093/nar/29.1.308 11125122 PMC29783

