# Supplementary material for: iMapSplice: Alleviating reference bias through personalized RNA-seq alignment
Source: PLoS One. 2018 Aug 10;13(8):e0201554. doi: 10.1371/journal.pone.0201554 (PMC6086400; doi:10.1371/journal.pone.0201554)
Supplement: S1 File — (DOCX) [file pone.0201554.s001.docx]

iMapSplice: alleviating reference bias through personalized

RNA-seq alignment

Supplementary Material

# iMapSplice algorithm and usage details

## Indexing

##
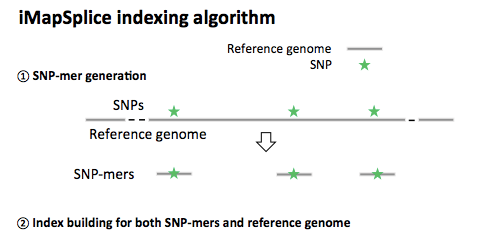


Figure 1. An overview of iMapSplice algorithm

iMapSplice indexing algorithm builds index files for both the reference genome and SNP-mers. There are two steps in the indexing algorithm: SNP-mer generation and indexing building. In the first step of SNP-mer generation, the genomic sequence segments carrying the SNPs are extracted and the SNPs are localized in the middle of the sequence segments. In the second step of indexing building, both the suffix array indices of the reference genome and SNP-mers are built. For the reference genome, we only need to index it for once, and its index files can be reused by other individuals. For SNP-mers, in addition to the suffix array indices, a map between SNP-mer coordinates and reference genome will also be built to facilitate converting aligned read segments in SNP-mers to reference genome coordinates. Differently, MapSplice does not generate SNP-mers and only builds index for the reference genome.

## Mapping


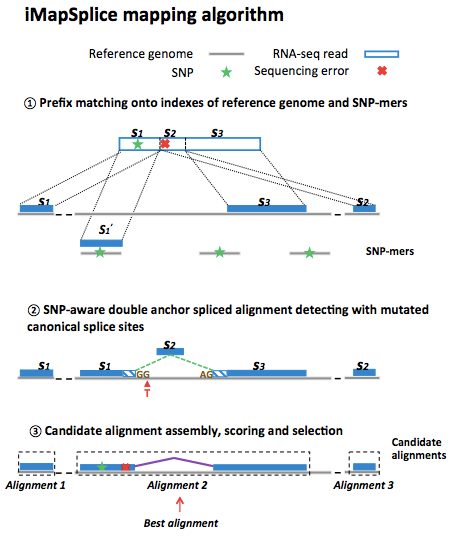


Figure 2. An overview of iMapSplice mapping algorithm

In the first step of iMapSplice mapping algorithm, it simultaneously maps RNA-seq reads to both the reference genome and SNP-mers using their suffix array indices. Then read segments mapped to SNP-mers will be converted to the reference genome coordinates and merged with other segments. Spliced alignment will be performed to bridge two adjacent read segments and detect the splice junctions. The last step in iMapSplice mapping algorithm assembles all the read segment alignments, scores the candidate read alignments, and selects the best one as the final output. iMapSplice differs from MapSplice in each of the three steps. In the first step, iMapSplice maps reads to both the reference genome and SNP-mers, while MapSplice will only map the reads onto the reference genome. In the second step, when performing spliced alignment detection, MapSplice relies on the reference genome only to search for the canonical splice site dinucleotides (e.g. GT-AG, GC-AG, and AT-AC). However, in iMapSplice, in addition to the reference bases, the alternative bases in SNP positions are also taken into consideration. This modification in algorithm improves iMapSplice’s capability in detecting the canonical splice junctions where SNPs are located in splice sites. In the last step, candidate alignments are scored on the total number of mismatches, spliced alignment, and mapped length. Different from MapSplice, iMapSplice removes the penalty of mismatches that can be attributed to SNPs.

## Schematic diagram of iMapSplice usage


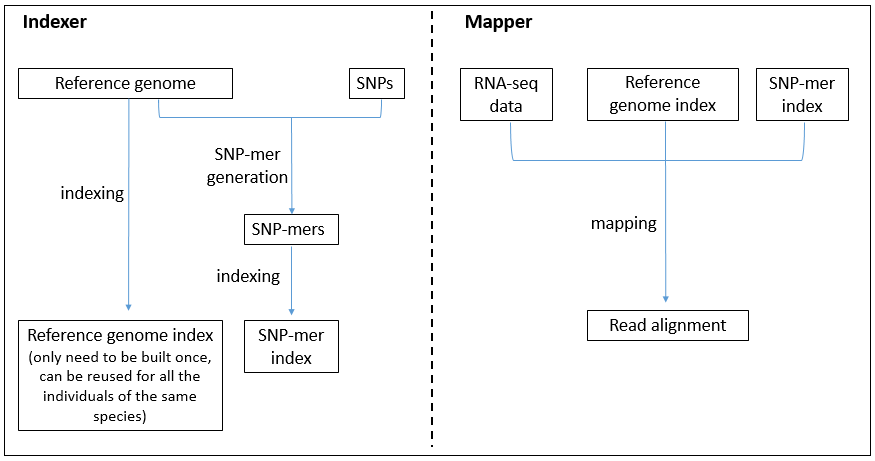


Figure 3. A schematic diagram of iMapSplice software usage.

iMapSplice software consists of two components: indexer and mapper. The input files for indexer are reference genome sequences (fasta) and a SNP table. The detailed format of SNP table can be found in https://github.com/LiuBioinfo/iMapSplice. Indexer first extracts SNP-mers based on the reference genome sequences and the provided SNP table, and then build suffix array indices for those SNP-mers. If the reference genome index has not been built yet, indexer can also be used to index the reference genome. The reference genome index can be reused for all the individuals of the same species. So it only needs to be built once. The second component mapper requires three input files: RNA-seq data (in fasta or fastq format), reference genome index, and SNP-mer indices. The output of mapper is read alignment file in SAM format [1]. Additionally, software usage instructions for different steps and modules (including command lines, input/output data formats, etc.) have been added to the code depository in GitHub: https://github.com/LiuBioinfo/iMapSplice.

# SNP-mer selection and its impact on iMapSplice performance

In iMapSplice, reads are simultaneously mapped to both reference genome and SNP-mers. Then read segments mapped to SNP-mers will be converted to the reference genome coordinates and merged with other segments. The aim of using SNP-mer is to rescue those read segments with SNPs inside as they will be missed by the reference genome based mapping. The SNP could be in any position of the read segment. The extreme case is when the SNP is located at the end (the first or the last position). So the SNP-mer should be long enough to accommodate it at either half of the sequence. Since the reference genome can be substantially long, only when the mapped read segment is longer than a certain length, we consider it as confidently aligned instead of a random match. Empirically, the current algorithms use a threshold from 16 to 25 [2,3]. So we suggest setting SNP-mer length at least 31 (allow a 16bp match at either half of the SNP-mer). At the same time, a SNP-mer too long is not necessary, as they will repeat the exact sequence from the reference genome. So we suggest setting SNP-mer no longer than twice the read sequence length (allow a full read sequence match at either half of the SNP-mer). To investigate the impact of SNP-mer length on iMapSplice performance, we conducted an experiment by applying iMapSplice with four different SNP-mer lengths (31, 51, 101, and 201) to human RNA-seq datasets from five individuals (NA12812, NA12749, NA07056, NA06994, and NA12275). The accumulated reference allelic ratio distributions of SNP positions with at least ten supporting reads are reported in the supplementary Table S2. As shown, all the four SNP-mer length settings exhibit symmetric distributions with respect to the reference allelic ratio of 0.5. At the same time, using SNP-mers of length 201 delivers the largest number of SNP positions with at least ten supporting reads although the superiority is very small. We believe this is due to the higher sequence matching possibilities between read sequence and SNP-mers when longer SNP-mers are used. Based on the analyses above, and the fact that most of the RNA-seq datasets today are no longer than 101bp, *k*_max_, *k*_phased_, and *k*_min_ are set as 201, 201, and 31 by default, and applied throughout all the experiments in this study. All the three parameters can be tuned by the users to optimize the performance.

# Simulated data information

Simulated RNA-seq data were generated using a popular tool Benchmarker for Evaluating the Effectiveness of RNA-Seq Software (BEERS) [4]. Firstly, BEERS randomly chose 30,000 mouse mRNA transcripts from a union set of 11 gene annotation datasets, including AceView, Ensembl, Geneid, Genscan, NSCAN, other RefSeq, RefSeq, SGP, Transcriptome, UCSC, and Vega. The *Mus musculus* genome assembly mm9 was used as the reference genome. Secondly, SNPs (substitutions), indels, and random sequencing errors were then introduced into the transcript sequences. In our experiments, we used two different variant and error profiles. The low error reads were generated assuming a substitution frequency of 0.001, indel frequency of 0.0005, and a base error frequency of 0.005. Corresponding rates in the high error reads were increased five-fold, 0.005, 0.0025, and 0.025 respectively. Then, the reads were randomly generated based on the randomly selected mRNA transcript sequences. For both error categories, we generated two RNA-seq datasets with different read lengths, 50bp and 100bp. Each dataset contained 20 million paired-end reads with the same insert length of 200 bp. After simulation, BEERS outputs the read sequences, the read sequence alignment ground truth, and the genomic variants (SNPs and indels) inserted into the mRNA transcripts.

# General Splice Junction Discovery Sensitivity and Specificity

Splice junctions detected by the aligners (iMapSplice, MapSplice [2], STAR [5], HISAT2 [6]) were compared with ground truth. Detected splice junctions were categorized as correct if they matched ground truth exactly at both the splice donor and acceptor sites. We evaluated aligner performance through comparing sensitivity and specificity of discovery of splice junctions with at least two supporting reads. Results are reported in Table 1. Sensitivity is the percentage of detected correct splice junctions among all the true junctions. Specificity is the fraction of detected correct splice junctions within all the detected junctions.

|  | **Low error 50bp** | | | **Low error 100bp** | | | **High error 50bp** | | | **High error 100bp** | | |
| --- | --- | --- | --- | --- | --- | --- | --- | --- | --- | --- | --- | --- |
|  | **Sensi** | **Speci** | **F-score** | **Sensi** | **Speci** | **F-score** | **Sensi** | **Speci** | **F-score** | **Sensi** | **Speci** | **F-score** |
| iMapSplice-unphased | 90.44 | 98.90 | 94.48 | 97.28 | 98.91 | 98.09 | 77.49 | 97.20 | 86.23 | 93.71 | 97.67 | 95.65 |
| MapSplice | 89.67 | 98.89 | 94.05 | 97.17 | 98.87 | 98.01 | 69.92 | 97.39 | 81.40 | 92.16 | 97.50 | 94.75 |
| STAR | 90.35 | 97.50 | 93.79 | 96.41 | 97.18 | 96.79 | 73.37 | 95.52 | 82.99 | 88.57 | 95.73 | 92.01 |
| HISAT2 | 89.99 | 97.88 | 93.77 | 96.40 | 97.52 | 96.96 | 61.35 | 96.96 | 75.15 | 74.99 | 95.74 | 84.10 |
| HISAT2 SNP | 90.13 | 98.18 | 93.98 | 96.35 | 97.70 | 97.02 | 65.36 | 97.02 | 78.10 | 79.09 | 98.52 | 86.53 |

Table 1: Sensitivity and specificity for splice junction discovery on simulated datasets.

# Impact of genomic variant frequency and sequencing error frequency on aligner performance

To further investigate how the aligners’ performance is impacted by genomic variant and sequencing error frequencies, we applied iMapSplice and MapSplice to another simulated dataset of 50bp reads with low frequency variants (SNP frequency of 0.001, indel frequency of 0.0005) and high frequency sequencing errors (error frequency of 0.025). Thus, the impact of sequencing errors can be assessed by comparing the results from the low error dataset used in the main text (low variant frequency and low sequencing error frequency) to the new dataset. Further, the impact of variants can be assessed by comparing the results in the new dataset to the high error dataset used in the main text (high variant frequency and high sequencing error frequency).

We assessed the aligners’ performance by counting the numbers of accurate unique alignments detected by each tool. The results of this dataset as well as the other two 50bp datasets described in the main text are shown in Table 2. In all the three datasets, iMapSplice reported more accurate unique alignment than MapSplice. Comparing the advantages of iMapSplice in different datasets, we observe a significant improvement of accuracy in the more challenging dataset (the one with higher variant frequency and higher sequencing error frequency).

In terms of the impact of variant and sequencing error frequency on the aligners’ performance, the five-fold increase of variant frequency leads to about 3.2 ~ 3.3 million drop in the numbers of reported accurate unique alignments (decreases from 18.9 to 15.7 million for iMapSplice and from 18.8 to 15.5 million for MapSplice), while the increase in sequencing error frequency leads to a much lower drop (decreases from 15.8 to 14.9 million for iMapSplice and from 15.5 to 13.5 million for MapSplice).

| Aligners | Accurate unique alignment number out of 20 million synthetic reads | | |
| --- | --- | --- | --- |
|  | Low variant frequency + low sequencing error frequency (low error data in main text) | Low variant frequency + high sequencing error frequency | High variant frequency + High sequencing error frequency (high error data in main text) |
| iMapSplice-unphased | 18,931,227 | 15,758,124 | 14,934,484 |
| MapSplice | 18,839,058 | 15,526,578 | 13,485,776 |

Table 2. The comparison of iMapSplice and MapSplice in terms of the number of reported accurate unique alignments out of 20 million synthetic reads in three simulated datasets of 50bp reads with different genomic variant and sequencing error frequency profiles.

# References

1. Li H, Handsaker B, Wysoker A, Fennell T, Ruan J, Homer N, et al. The Sequence Alignment / Map format and SAMtools. Bioinformatics. 2009;25: 2078–2079. doi:10.1093/bioinformatics/btp352

2. Wang K, Singh D, Zeng Z, Coleman SJ, Huang Y, Savich GL, et al. MapSplice: Accurate mapping of RNA-seq reads for splice junction discovery. Nucleic Acids Res. 2010;38. doi:10.1093/nar/gkq622

3. Philippe N, Salson M, Commes T, Rivals E. CRAC: An integrated approach to the analysis of RNA-seq reads. Genome Biol. 2013;14. doi:10.1186/gb-2013-14-3-r30

4. Grant GR, Farkas MH, Pizarro AD, Lahens NF, Schug J, Brunk BP, et al. Comparative analysis of RNA-Seq alignment algorithms and the RNA-Seq unified mapper (RUM). Bioinformatics. 2011;27: 2518–2528. doi:10.1093/bioinformatics/btr427

5. Dobin A, Davis CA, Schlesinger F, Drenkow J, Zaleski C, Jha S, et al. STAR: Ultrafast universal RNA-seq aligner. Bioinformatics. 2013;29: 15–21. doi:10.1093/bioinformatics/bts635

6. Kim D, Langmead B, Salzberg SL. HISAT: a fast spliced aligner with low memory requirements. Nat Methods. 2015;12: 357–60. doi:10.1038/nmeth.3317
